# Acute Bilateral Lower Limb Ischemia Mimicking Neurological Emergency: A Case of Leriche Syndrome

**DOI:** 10.7759/cureus.96397

**Published:** 2025-11-08

**Authors:** Bharath Sundaramoorthy, Muthusankar Sudalaimuthu, Suresh Kumar Gopala Pillai

**Affiliations:** 1 General Medicine, Government Thoothukudi Medical College, Thoothukudi, IND; 2 Emergency Medicine, Swansea Bay University Health Board, Swansea, GBR; 3 Emergency Medicine, Morriston Hospital, Swansea, GBR

**Keywords:** acute limb ischemia, aortoiliac thrombosis, bilateral leg weakness, ct angiography, emergency medicine, leriche syndrome, reperfusion time, vascular emergency

## Abstract

Leriche syndrome, a rare manifestation of chronic aorto-iliac occlusive disease, may occasionally present acutely with bilateral lower limb ischemia, mimicking neurological emergencies such as cauda equina syndrome. We report a 70-year-old man presenting with sudden-onset bilateral lower limb weakness and numbness. Examination revealed cold, pulseless lower limbs. CT angiography demonstrated extensive thrombosis from the descending thoracic aorta to the iliac arteries, with complete occlusion of the right superficial femoral artery. The patient underwent urgent open embolectomy of the femoral and subclavian arteries, aorto-bifemoral bypass via axillary artery anastomoses, and bilateral lower limb fasciotomies. Although perfusion was successfully restored in the left lower limb, irreversible ischemia in the right leg necessitated an above-knee amputation. This case highlights the importance of early recognition of acute aorto-iliac thrombosis, rapid vascular imaging, and coordinated multidisciplinary intervention to optimize outcomes in this rare yet critical vascular emergency.

## Introduction

Leriche syndrome, also known as chronic aorto-iliac occlusive disease, is a rare manifestation of progressive atherosclerotic narrowing of the distal abdominal aorta and iliac arteries. It is classically described by the triad of intermittent claudication of the lower limbs, absent or diminished femoral pulses, and erectile dysfunction [1]. Typically, the disease develops insidiously over years, and patients often adapt to gradually worsening symptoms, making early recognition challenging. In some instances, however, the syndrome may present acutely due to sudden aorto-iliac thrombosis, leading to abrupt bilateral lower limb ischemia. Such acute presentations are uncommon but carry a significantly higher risk of limb loss and mortality compared to chronic disease [2,3].

Acute Leriche-type occlusion can closely mimic neurological emergencies such as spinal cord compression, transverse myelitis, or cauda equina syndrome, particularly when presenting with sudden bilateral lower limb weakness, numbness, or paresthesia [3]. This overlap often results in initial misdiagnosis, potentially delaying life- and limb-saving intervention. A careful physical examination, particularly assessment of limb temperature, color, capillary refill, and femoral pulses, is therefore essential in distinguishing vascular from neurological etiologies. Rapid imaging, most commonly with computed tomography (CT) angiography, plays a pivotal role in confirming the diagnosis, delineating the extent of thrombosis, and guiding urgent surgical or endovascular intervention [4,5].

Furthermore, older or sexually inactive patients may not provide a history of erectile dysfunction, which traditionally forms part of the classic triad. In such cases, reliance on vascular signs and imaging findings becomes critical for timely recognition. Studies have demonstrated that delays in diagnosis or reperfusion beyond 24 hours are strongly associated with higher rates of limb loss and mortality [6]. Therefore, maintaining a high index of suspicion, performing thorough vascular assessment, and coordinating multidisciplinary care between emergency, radiology, and vascular teams are paramount in optimizing outcomes in these rare but severe presentations [4-6].

## Case presentation

A 70-year-old man with a history of hypertension and no history of smoking presented to the emergency department with a history of sudden-onset bilateral lower limb weakness and loss of sensation below the waist for less than 24 hours. The acute symptoms occurred at rest, although he reported a two-week history of intermittent right calf cramping on exertion that had resolved spontaneously. There was no trauma, incontinence, or preceding neurological illness. On arrival, his vital signs were the following: BP 140/80 mmHg, HR 101/min, RR 16/min, temp 36.8°C, and SpO₂ 97% on room air.

Neurological examination revealed reduced motor power in the left leg (4/5) and variable power in the right leg, with 0/5 at the ankle. Bilateral lower limb reflexes were absent, and sensation was globally reduced below the waist. Both lower limbs were cold with prolonged capillary refill, and femoral, popliteal, posterior tibial, and dorsalis pedis pulses were absent bilaterally. Bedside Doppler assessment confirmed the absence of distal arterial signals in both lower limbs. Bladder assessment was performed to help differentiate neurological causes such as spinal cord compression or cauda equina syndrome. The pre-void volume was 305 mL, and the post-void residual was 80 mL, with normal per rectal tone, indicating preserved bladder function (Table 1).

**Table 1:** Clinical, neurological, and vascular findings on presentation with emphasis on red-flag signs for acute limb ischemia

Parameter	Findings on arrival	Clinical significance
Blood pressure	140/80 mmHg	Normotensive; perfusion pressure preserved
Heart rate	101/min	Mild tachycardia; early ischemic stress response
Respiratory rate	16/min	Normal
Temperature	36.8°C	Afebrile; no systemic infection
SpO₂	97% on room air	Adequate oxygenation
Motor power	Left leg: 4/5; right leg: variable, 0/5 at ankle	Severe ischemic impairment, right worse; red flag for urgent intervention
Reflexes	Bilateral lower limb reflexes absent	Could mimic neurological pathology; ischemic neuropathy possible
Sensation	Globally reduced below the waist	Significant bilateral involvement; red flag
Peripheral vascular	Both lower limbs cold; capillary refill prolonged; femoral, popliteal, posterior tibial, and dorsalis pedis pulses absent bilaterally	Strong evidence of acute arterial occlusion; critical red flag
Bladder function	Pre-void 305 mL; post-void 80 mL; per rectal tone intact	Partial bladder emptying; no overt spinal cord involvement

**Figure 1:**
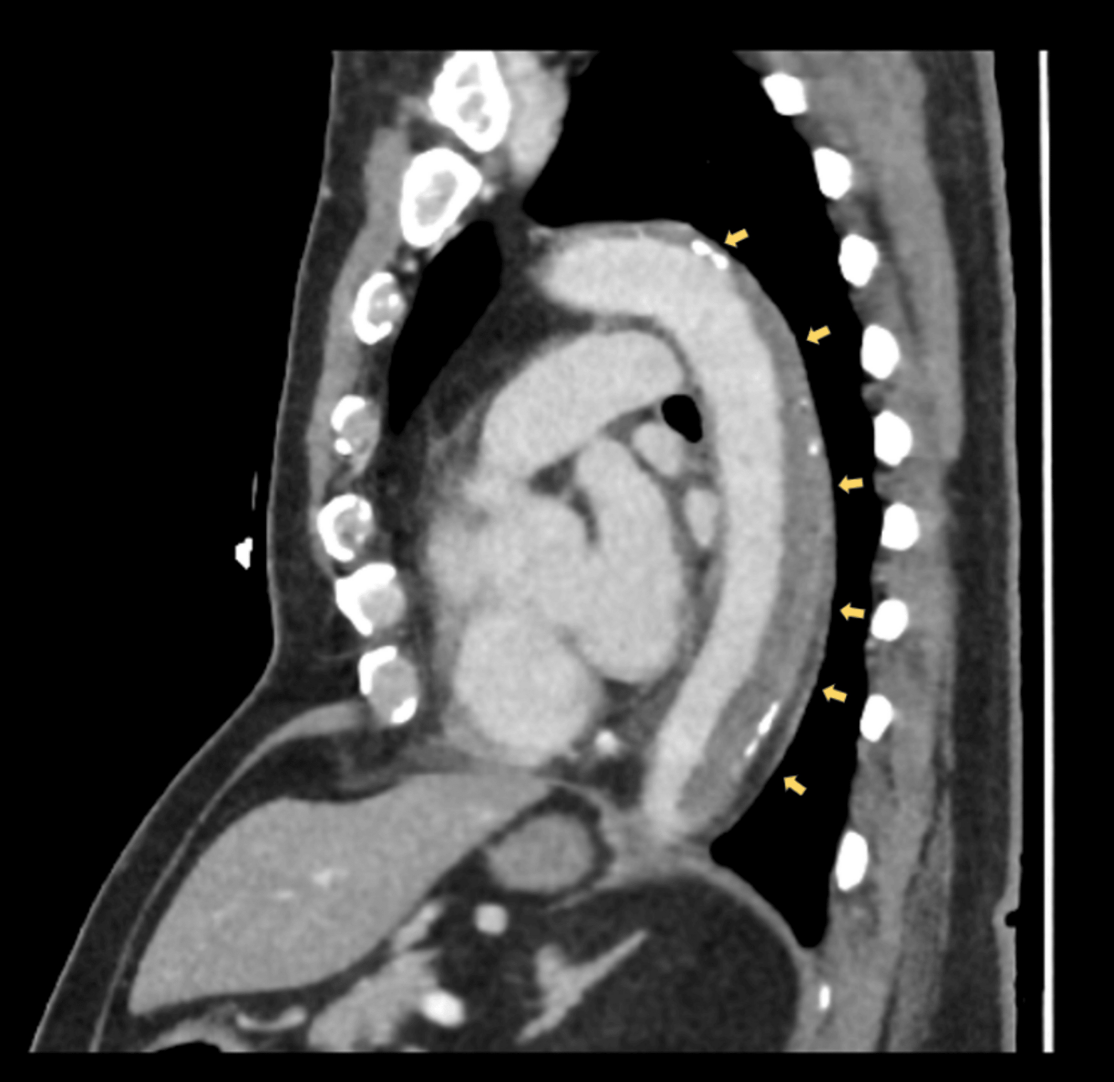
Sagittal CT angiography of the thoracic aorta. Demonstrates mural thrombus along the posterior wall of the descending thoracic aorta (yellow arrows), occupying approximately 50% of the aortic lumen. CT: computed tomography

**Figure 2:**
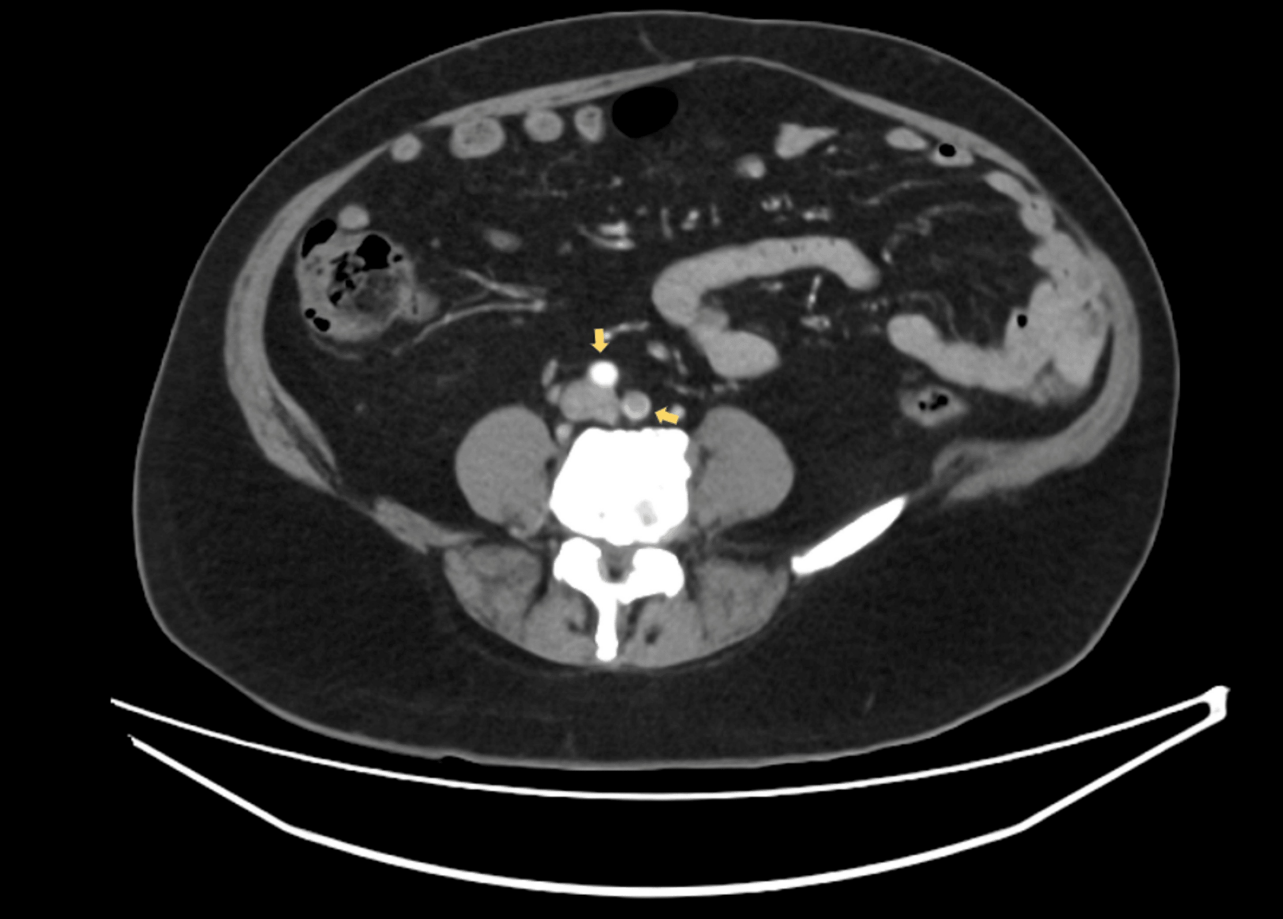
Axial CT angiography of the abdominal aorta and iliac arteries. Demonstrates thrombus within the infrarenal abdominal aorta (yellow arrows) extending into the origins of the common iliac arteries, consistent with aorto-iliac occlusion. CT: computed tomography

**Figure 3:**
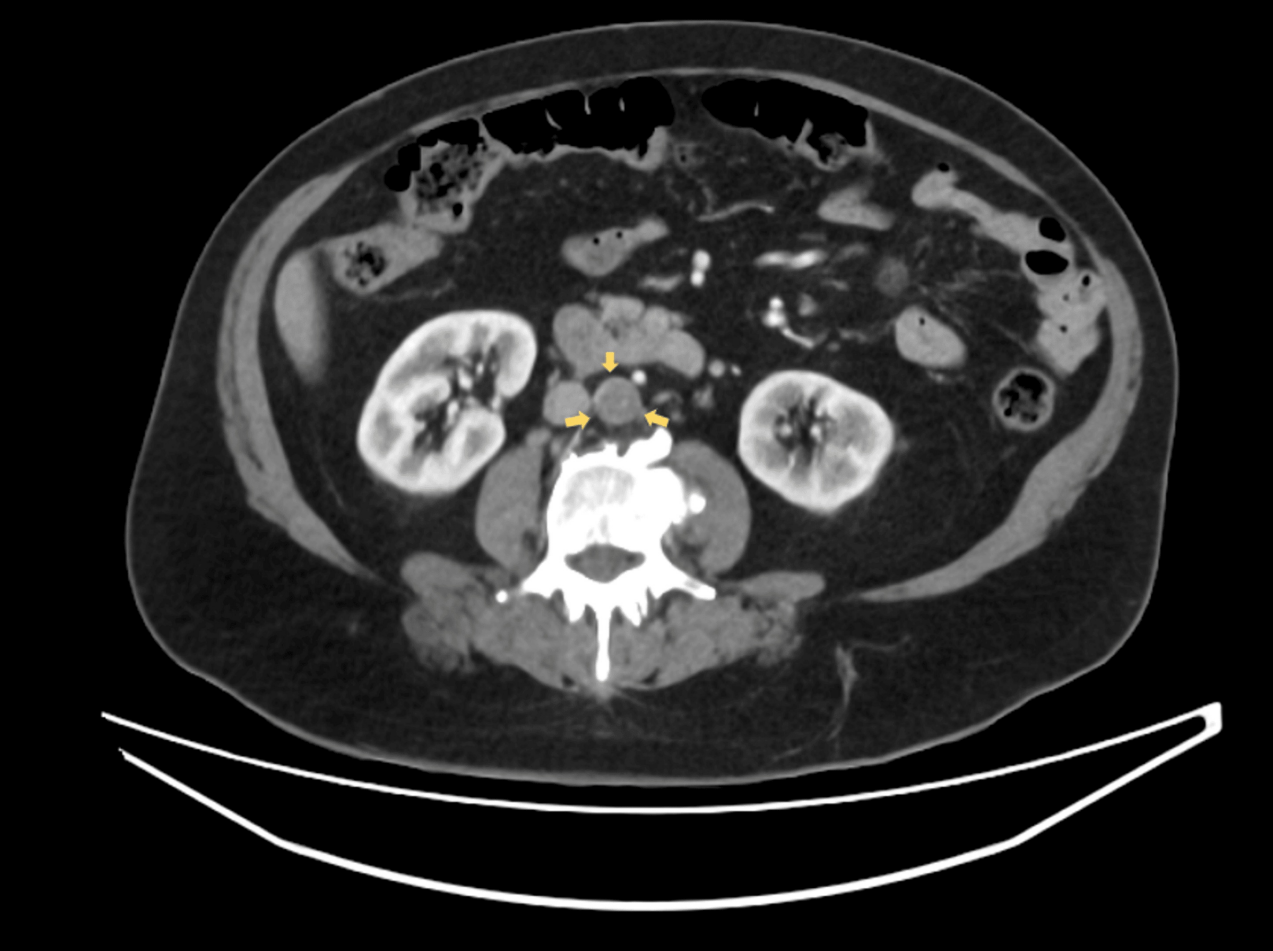
Axial CT angiography of the abdominal aorta and iliac arteries. Demonstrates near-total occlusion of the infrarenal abdominal aorta with intraluminal thrombus (yellow arrows) extending into the origins of both common iliac arteries. CT: computed tomography

**Figure 4:**
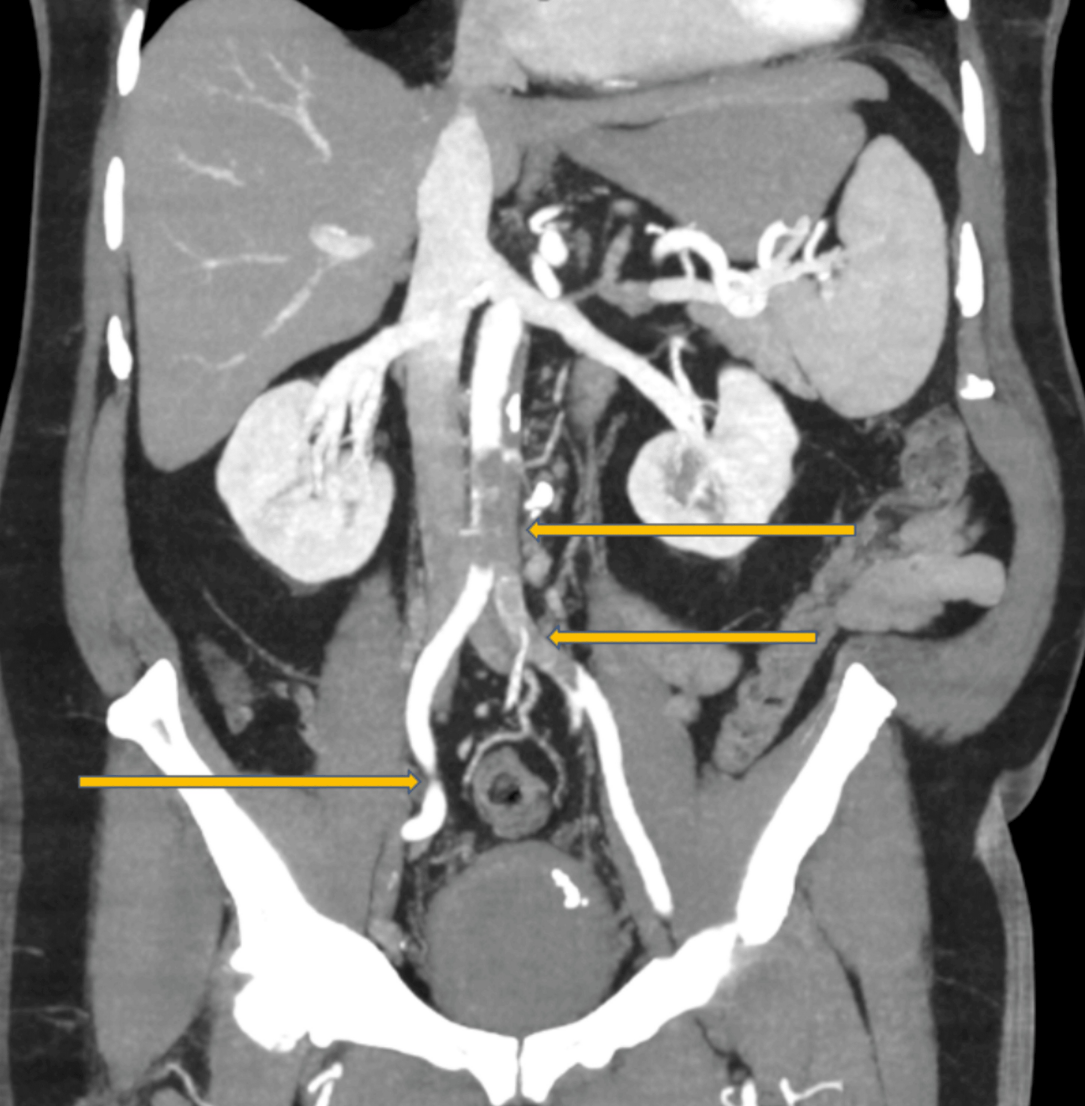
Coronal CT angiography of the abdominal aorta and iliac arteries demonstrating extensive intraluminal thrombus (yellow arrows) extending from the infrarenal abdominal aorta into the bilateral common iliac arteries. The thrombus causes near-total luminal occlusion with markedly reduced distal contrast opacification, consistent with acute aorto-iliac thrombosis. CT: computed tomography

**Figure 5:**
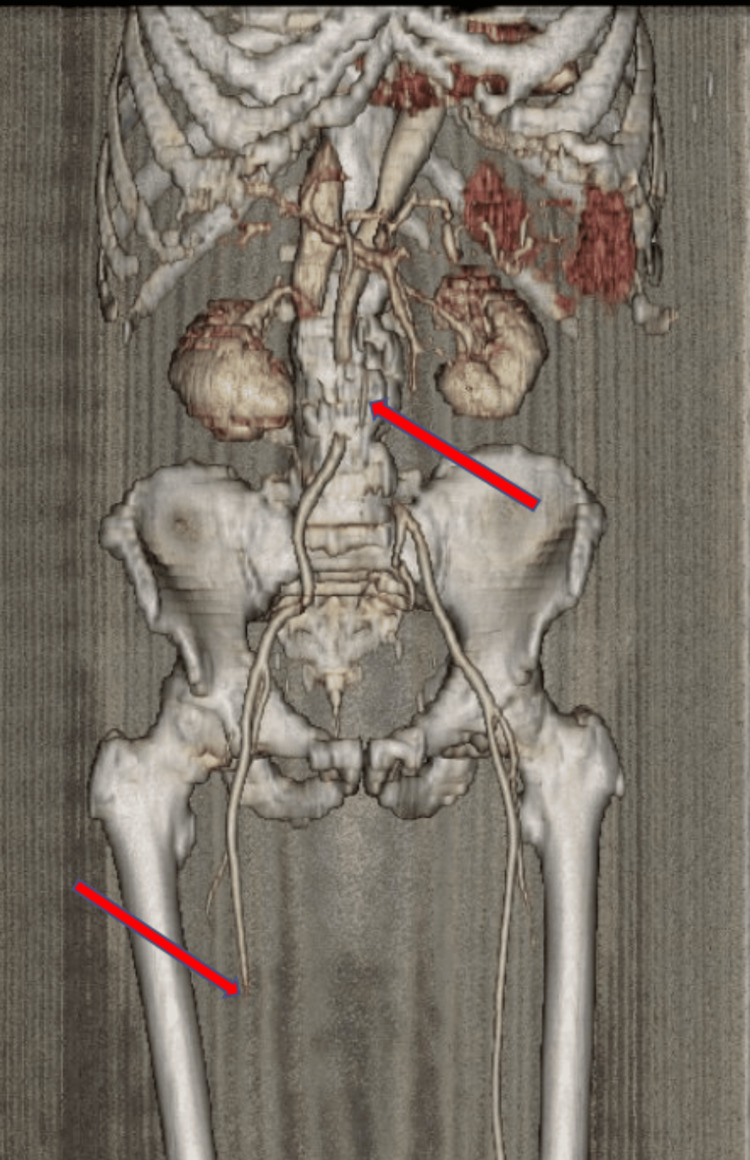
3D reconstructed CT angiography of the aorto-iliac and femoral arteries. Demonstrates extensive thrombus extending from the abdominal aorta into the bilateral iliac arteries, with poor distal runoff and complete occlusion of the right superficial femoral artery (red arrows). 3D: three dimensional, CT: computed tomography

Electrocardiogram revealed sinus tachycardia without acute ischemic changes. An ankle-brachial index was not performed due to the acute, severe nature of limb ischemia with absent distal pulses, as urgent imaging and revascularization were required. A CT angiography demonstrated extensive thrombus extending from the descending thoracic aorta to the abdominal aorta and bilateral iliac arteries, with poor distal runoff, particularly on the right side. Sagittal imaging of the thoracic aorta revealed mural thrombus along the posterior wall occupying approximately 50% of the lumen (Figure 1). Axial images of the abdominal aorta and iliac arteries demonstrated near-total occlusion of the infrarenal aorta extending into both common iliac arteries, with complete occlusion of the right superficial femoral artery (Figures 2-4). Three-dimensional reconstruction provided a detailed view of thrombus distribution and poor distal perfusion (Figure 5).

The vascular surgery team was consulted urgently, and approximately five hours elapsed from presentation to theater, allowing for a coordinated multidisciplinary assessment. The patient underwent emergency open embolectomy of the femoral and subclavian arteries, followed by an aorto-bifemoral bypass via axillary artery anastomoses. Bilateral lower limb fasciotomies were performed to prevent compartment syndrome. The procedure was completed without intraoperative complications. The total time from symptom onset to reperfusion was approximately 29 hours, including the 24-hour pre-hospital period of intermittent symptoms.

Postoperatively, perfusion of the left limb was restored via collateral circulation, and it remained viable. In contrast, the right limb failed to recover perfusion despite immediate revascularization, necessitating a right above-knee amputation. The patient subsequently underwent cardiovascular risk factor optimization and rehabilitation, gradually regaining functional independence.

## Discussion

Acute Leriche-type aorto-iliac thrombosis is an uncommon but potentially devastating vascular emergency. While classical Leriche syndrome is typically chronic and characterized by the triad of claudication, absent femoral pulses, and erectile dysfunction [1], acute presentations may mimic neurological conditions such as cauda equina syndrome or spinal cord infarction [2,3]. Similar cases in the literature report initial misdiagnosis as cauda equina or spinal cord pathology, highlighting the importance of thorough vascular assessment [7,8]. This overlap can delay diagnosis and definitive management, increasing the risk of limb loss or mortality.

Acute aorto-iliac thrombosis may present with sudden bilateral leg weakness, sensory loss, absent reflexes, and bladder changes, features that can resemble neurological syndromes. Unlike cauda equina syndrome, vascular occlusion produces cold, pale, pulseless limbs, whereas cauda equina usually presents with warm limbs and preserved pulses. In this patient, the absence of distal pulses confirmed by bedside Doppler established a vascular cause, prompting urgent revascularization. Timely recognition and intervention are crucial in reducing the risk of limb loss and other complications.

In this case, bedside findings (cold limbs, absent femoral pulses, and prolonged capillary refill) guided clinicians toward a diagnosis of vascular origin. Hypertension may have contributed through endothelial injury and atherothrombotic changes, predisposing to in situ thrombosis.

CT angiography remains the gold standard for evaluating aorto-iliac occlusion, providing detailed visualization of the aorta, iliac arteries, and distal runoff, which is essential for planning surgical or endovascular management [1,5]. In this patient, imaging revealed extensive thrombosis from the descending thoracic aorta to the iliac arteries, illustrating the severity of bilateral limb ischemia.

Management requires urgent revascularization. Surgical options include thrombectomy and aorto-bifemoral or axillobifemoral bypass, while endovascular techniques, such as catheter-directed thrombolysis and aspiration thrombectomy, are increasingly employed [4,5]. Outcomes depend heavily on time to reperfusion, with delays beyond 24 hours associated with higher rates of limb loss and mortality [6]. Despite timely intervention, irreversible ischemic injury may still necessitate amputation.

This case underscores key lessons: clinicians should maintain a high index of suspicion for vascular occlusion in patients with acute bilateral leg weakness, perform rapid CT angiography, and involve a multidisciplinary team promptly. Even when classic features such as erectile dysfunction are absent or unknown, diagnoses can be reliably made based on vascular examination and imaging findings [1-3].

Our case contributes to the literature by illustrating a rare acute presentation that mimics neurological disease, emphasizing the importance of prompt recognition of vascular signs and imaging confirmation to guide timely intervention, and reinforcing the recommendations from previous studies on rapid assessment and multidisciplinary management to reduce limb loss and mortality [4,7,8].

## Conclusions

Acute Leriche-type aorto-iliac thrombosis is a rare but critical vascular emergency that can mimic neurological conditions such as cauda equina syndrome. In this case, sudden bilateral lower limb weakness, absent pulses, cold extremities, bladder findings, and CT angiography-confirmed thrombus were key in establishing the vascular etiology and guiding timely intervention. The case highlights the importance of thorough vascular assessment, rapid imaging, and multidisciplinary management to optimize outcomes and minimize limb loss. While this report focuses on a single patient, it highlights essential clinical features and diagnostic strategies that can help clinicians recognize similar atypical presentations and support early diagnosis and intervention.
